# Effect of cadmium bioavailability in food on its compartmentalisation in carabids

**DOI:** 10.1007/s10646-017-1851-y

**Published:** 2017-09-13

**Authors:** Agnieszka J. Bednarska, Zuzanna M. Świątek, Karolina Paciorek, Natalia Kubińska

**Affiliations:** 1 Institute of Nature Conservation, Polish Academy of Sciences, Mickiewicza 33, 31-120 Kraków, Poland; 2 Institute of Environmental Sciences, Jagiellonian University, Gronostajowa 7, 30-387 Kraków, Poland

**Keywords:** Metal, Sequestration, Bioavailability, Toxicokinetic, Food web transfer

## Abstract

Metals assimilated by organisms are sequestered in various compartments and some forms are more stable than others. Sequestration mechanisms used by invertebrates to detoxify metals and prevent interaction with important biomolecules include metal binding to proteins and other ligands, and storage in inorganic granules. The rate and extent at which metal concentrations in different compartments respond to metal concentrations in food and food characteristics has not received much attention, despite being of great relevance. We performed an experiment on the carabid beetle *Pterostichus oblongopunctatus* exposed to Cd via food made of ground mealworm (*Tenebrio molitor*) larvae, either reared on Cd contaminated medium or artificially spiked after grinding with CdCl_2_ solution. Thus, in both cases we used the same type of food, differing only in the soluble Cd pool available to the predators, represented by *P. oblongopunctatus*. Subcellular compartmentalisation of Cd into organelles, heat-sensitive and heat-stable proteins (the first supernatant, S1 fraction), cellular debris (the second supernatant, S2 fraction) and metal-rich granules (G fraction) was checked a few times during the contamination (90 d) and decontamination (24 d) phases in a toxicokinetic experiment by using different centrifugation steps. The results showed no effect of the type of food (naturally, Cd-N, vs. artificially contaminated with Cd, Cd-A) on Cd sequestration kinetics in *P. oblongopunctatus*, but the amount of Cd sequestered in the S1 and G fractions were in general higher in the Cd-A than the Cd-N treatment, indicating that Cd transfer in the food web depends on the speciation of the metal in the food. The proportional distribution of Cd over different fractions was, however, similar in beetles fed both food types. Most of the accumulated Cd in the beetles existed as fraction S1 (*ca*. 35%), which is important for the transfer of metals to higher trophic levels in a food web.

## Introduction

One of the major challenges in assessing potential effects of toxicants on organisms is predicting the internal active concentration of toxic chemicals in the body and/or target organs. Toxic effects estimated on the basis of internal body/tissue concentrations rather than on external exposure (e.g. concentration in food) are often far less variable among species, different chemicals with similar mode of action, and different environmental conditions (McElroy et al. 2010). In organisms exposed to high metal concentrations, physiological acclimation, for example through shifts in toxicokinetics (TK), may help to overcome potential chemical stresses, especially during prolonged exposure to metals under sublethal concentrations. Indeed, several experiments on terrestrial invertebrates revealed that during prolonged metal exposure, animals are able to maintain metal concentration unchanged in their bodies (Kramarz 1999) or to even decrease it (Bednarska et al. 2011). The usefulness of TK parameters (i.e. assimilation and elimination rate constants) derived from single-concentration laboratory experiments for predicting metal concentrations in organisms in actual polluted environments has been, however, questioned recently (Bednarska et al. 2015). After testing the toxicokinetic parameters for a range of exposure concentrations of Zn and Cd in crickets *Gryllus assimilis*, Bednarska et al. (2015) concluded that there is no metal-specific assimilation or elimination rates. Not only exposure concentration in food and/or “toxic power” of a metal, but also a type of food used in TK experiments can potentially affect the TK parameters. The impact of the bioavailability of a metal in food on TK and sequestration processes, however, has not been studied so far for orally exposed animals.

Metals are present in food in different forms, and the main two pools are metal ions bound to the solid phase and the soluble metal pool. Their ratio can differ depending on the food type. Some data on how different fractions of metals may affect TK processes exist for invertebrates which were exposed to metals through soil (Vijver et al. 2005), but there is no similar TK data for orally exposed terrestrial invertebrates, and in particular carnivores. However, the data on the effect of subcellular partitioning of Cd in plants on the trophic transfer of Cd to the isopod *Porcellio dilatatus* measured after 28 d of exposure (Monteiro et al. 2008), highlight that the subcellular Cd distribution in food may have an important impact on trophic transfer to the consumer.

In most TK experiments with oral exposure, an artificial food is used as such food is easy to contaminate with the toxicant of interest. However, using artificial food may lead to significant inaccuracies when results obtained in laboratory experiments need to be extrapolated to the field situation. To give an example, in their study on Zn and Cd kinetics in the ground beetle, *P. oblongopunctatus*, Lagisz et al. (2005) observed metal elimination already in the uptake phase, which did not allow to fit the one-compartment model to their data. Their results were especially surprising, since uptake and elimination kinetics consistent with expectations of the classic one-compartment model were observed for the same metals in the closely related carabid *P. cupreus* (Kramarz 1999). One may suppose that the differences observed between the results for *P. oblongopunctatus* (Lagisz et al. 2005) and for *P. cupreus* (Kramarz 1999) were not (or not only) due to the use of different exposure concentrations and/or beetle species, but rather due to the use of different medium to feed the beetles. Lagisz et al. (2005) offered artificial food (dried chicken meat mixed with metal-salt solution) to the beetles, whereas Kramarz (1999) fed the beetles with housefly larvae reared on artificial medium contaminated with metals. The latter method seems to better resemble the actual feeding habits of the ground beetles, and bioavailability of the metals might differ between those two diets.

For metals, internal compartmentalisation determines what fraction of the metal is present in the body in a metabolically available form (Rainbow 2002). Sequestration mechanisms used by invertebrates to detoxify metals and prevent interaction with important biomolecules include metal binding to proteins and other ligands and storage in inorganic granules (Hopkin 1989). The storage of metals as an energy-efficient method of detoxification and subsequent excretion with faeces was already suggested by Simkiss (1976, 1977). Thus, it is apparent that similarly to the external concentration, only a portion of total metal body burden is biologically available for interaction with sites of toxic action. The methods of metal sequestration at a sub-cellular level have been summarised by Vijver et al. (2004), who found that it is very likely that the differences in metal sequestration have an impact on the ability of metal excretion by earthworms. We used the procedure which isolates three metal fractions: (1) metal-rich granules, (2) cytosolic fraction (a fraction combining microsomes and lysosomes, mitochondria, metallothioneins and heat sensitive proteins), and (3) cellular debris (a fraction consisting of cell membranes, tissue fragments and intact cells). A similar procedure has been previously used for such terrestrial invertebrates as earthworms (Beaumelle et al. 2015, Vijver et al. 2007), snails (Gimbert et al. 2008) and beetles (Bednarska and Świątek 2016).

Several studies have investigated the effects of metal distribution within prey on its assimilation by predators in aquatic food chains (e.g. Wang and Rainbow 2006). As far as terrestrial invertebrates are concerned, there are some data on effects of chemical partitioning of metals in exposure medium on its bioavailabilty (Peijnenburg et al. 1999), TK and internal sequestration (Vijver et al. 2004), but only for invertebrates exposed via soil and soil solution. However, such data obtained for soil dwelling organisms cannot be easily extrapolated to epigeic insects, which at least in the adult stage are exposed by uptake of contaminated food. This particularly relates to a range of carnivorous species such as *P. oblongopunctatus*. Moreover, the rate and extent at which metal concentrations in different fractions respond to metal concentrations in food and food characteristics over time has not received much attention, despite being of great relevance.

The aim of this study was to investigate the kinetics of cadmium and its compartmentalisation in the ground beetle *Pterostichus oblongopunctatus* (Coleoptera: Carabidae) exposed to the metal via food made of *Tenebrio molitor* larvae, either artificially contaminated with metal-salt solution or reared on contaminated medium (such foods are supposed to differ in the soluble metal pool available for predators, represented by *P. oblongopunctatus*). To understand the toxicokinetics processes better, the toxicokinetics models were fitted to different ‘biochemical’ compartments with different affinity for metals, i.e., cytosolic fraction, cellular debris and metal-rich granules.

## Materials and methods

### Studied species

The experiment was conducted on the ground beetle, *Pterostichus oblongopunctatus* (Coleoptera: Carabidae). Ground beetles as a group are important for their effectiveness in consuming prey insects, many of which are pest herbivores. They also occupy an important position in the food web, being food for amphibians, reptiles, birds and mammals (Lövei and Sunderland 1996). *P. oblongopunctatus* can be considered a typical representative of the epigeic carnivorous insects. The individuals were collected with pitfall traps from uncontaminated sites near Pilica, southern Poland, in April 2014. Female beetles were not used in this study because *P. oblongopunctatus* mate during April/May (Brunsting 1981), as such, there was a high probability that some of the caught females were fertilised, and this can influence toxicokinetics. To minimize an influence on the toxicokinetics, only male beetles were used. The males were kept for 4 weeks in a climatic chamber at 20 °C and 75% relative humidity (RH) under a light:dark regime 16 h:8 h, in plastic boxes (*ca*. 500–2000 ml), with perforated lids, 10 to 25 individuals per box. The boxes were filled to approximately 1 cm with moistened peat, and 1–2 pieces of wet clay pot were placed in each box to keep the moisture and provide a shelter for the beetles. During this 4-week acclimation period, the beetles were fed *ad libitum*, three times a week, with artificial food made of ground mealworms (*Tenebrio molitor* obtained from a pet shop in Kraków, Poland) mixed with ground apple (Nestlé, Gerber) at 9:1 ratio and supplemented with *ca*. 1 g sodium benzoate (C_7_H_5_NaO_2_; Fluka, Germany) per kilogram food as a preservative. Two weeks before the experiment the wet peat was replaced with wet sand (Grudzień Las Sp. z.o.o., Poland) for practical reasons.

## Experimental design

### Toxicokinetic experiment

The classical TK experiment in which beetles were first fed ad libitum with contaminated food (uptake phase, here 90 d) and afterwards offered uncontaminated food (decontamination phase, here 24 d) was performed. The beetles were fed either food ‘naturally’ contaminated with Cd (Cd-N)—the food made of ground mealworms (*Tenebrio molitor*) reared on contaminated flour (nominal concentration of 300 mg kg^−1^) and then mixed with ground apple at 9:1 ratio, or the food artificially contaminated with Cd (Cd-A)—the food made of ground mealworms kept on uncontaminated flour and spiked with metal-salt solution (CdCl_2_ × 2.5H_2_O, POCH, Poland) to obtain the same Cd concentration as the one measured in the ‘naturally’ contaminated mealworms and then mixed with ground apple at 9:1 ratio. Thus, the same type of food differing only in chemical partitioning of metal in the food was used. The control treatment with uncontaminated food offered throughout the experiment was also included. The beetles were fed and their survival was checked three times a week.

Prior to the experiment, the beetles were weighed to the nearest 0.0001 g (Radwag AS/C/2, Poland) and placed individually in 30-ml plastic boxes filled to *ca*. 1/4 with moistened sand (Grudzeń Las Sp. z o. o., Poland). In each box a piece of wet clay pot was placed to provide a shelter for the beetles. The beetles were randomly allocated to treatments (93 to Cd-N, 91 to Cd-A and 55 to control treatment) and were sampled before starting the exposure (d 0) and after 2, 4, 6, 10, 18, 26, 46, 66 and 90 d of exposure (uptake phase) and at d 92, 96, 108, 114 (decontamination phase) from each Cd treatment (Cd-N, Cd-A). Ten beetles were sampled at d 0 and five were sampled from each Cd treatment until the 18 d, but later the number of sampled beetles had to be reduced to four or three because of the mortality. The control beetles were fed uncontaminated food throughout the experiment (120 d) to check their survival in comparison with Cd-exposed beetles, and were sampled to monitor for background Cd levels in their bodies at d 6, 26, 46, 66, 90, 96 and 114. The sampled beetles were starved for 24 h to void their gut of the food remnants, washed with deionised water to remove all remnants of food from their body surface, weighed to the nearest 0.0001 g (Radwag AS/C/2, Poland) and killed by freezing at −20 °C.

### Subcellular fractionation

The fractionation procedure based on a number of centrifugation steps and chemical treatments was used to isolate metal-rich granules and tissue fragments from intracellular (nuclear, mitochondrial, and microsomal) and cytosolic fractions (i.e. metallothioneins and heat sensitive proteins) as described by Vijver et al. (2006), but optimised for beetles (Bednarska and Świątek 2016). In brief, the legs, head with thorax, elytra and wings i.e. the most heavily sclerotized parts of exoskeleton, were carefully removed from the beetle using forceps and a scalpel and placed in a glass tube (fraction C). The remaining body parts of the beetle (i.e., all tissues apart from the abovementioned most heavily sclerotized parts of exoskeleton, but including the anterior gut which was dissected from the thorax) were homogenised with a tissue homogeniser (Gen-Bio PRO200) in 500 µL of ice-cold 0.01 M Tris-HCl buffer (pH 7.5, Sigma-Aldrich, USA). Then, the homogenate was centrifuged at 10 000 *g* for 30 min at 4 °C to separate the supernatant (fraction S1) from the pellets. The pellets were heated at 100 °C for 2 min, then 250 µL of 1 M NaOH (POCH, Poland) was added, and the fraction was hydrolysed for 1 h at 70 °C. After centrifuging at 10 000 *g* for 10 min at 20 °C, the supernatant consisting of metal ions bound to cellular debris (fraction S2) was separated from the pellets (granular fraction, G). The pellets were resuspended in 600 µL of 0.2% HNO_3_ (69.0–70.0%, INSTRA-Analysed, Baker, Germany). To control for possible contamination during the homogenisation and fractionation procedures, the blanks were conducted using 500 µL of 0.01 M Tris-HCl buffer. All samples were stored at 4 °C until they were analysed for metal concentrations.

### Chemical analysis

To analyse Cd concentrations in the flour used to culture the mealworm larvae, in the mealworms and in the food made of the ground mealworms, three samples of flour, ground mealworms and food per treatment were dried at 105 °C for 24 h and weighed to the nearest 0.001 g (Radwag AS/C/2, Poland) before digesting in 1.5 mL boiling HNO_3_ (69.0–70.0%, INSTRA-Analysed, Baker, Germany) and then diluting to 5 ml with 0.2% HNO_3_. To analyse Cd in subcellular fractions, the samples were placed in glass tubes and dried at 105 °C for 12 h before digesting in 300 µL of boiling HNO_3_. After complete digestion, the excess of acid was evaporated and the samples were diluted to 1 ml with 0.2% HNO_3_. Cd concentrations in the flour and food were measured using a flame atomic absorption spectrophotometer (Perkin-Elmer AAnalyst 200; detection limit: 0.011 mg L^−1^), and a graphite furnace atomic absorption spectrophotometer (Perkin-Elmer AAnalyst 800; detection limit: 0.024 µg L^−1^) was used to measure Cd concentration in samples after subcellular fractionations. To check the analytical precision, three blanks (acid only) and three samples of reference material (fish liver—C*ertified Reference Material Dolt-4 Dogfish Liver*, National Research Council of Canada) were run with the samples. The certified concentration in the reference material was 24.3 ± 0.8 mg kg^−1^ and the average Cd concentrations measured in the reference material used for the flour and food analysis was 22.1 ± 0.55 mg kg^−1^ and for and subcellular fractions analysis was 26.7 ± 7.11 mg kg^−1^. The results were not corrected for recovery. The concentrations of Cd in the flour and food were expressed in mg kg^−1^ dry weight (d.w.), and the amount of Cd found in each fraction was normalised by dividing it by the fresh body weight of a beetle and expressed in mg kg^−1^ wet weight (w.w.).

### Statistical analysis

The pattern of changes in Cd concentration (*C*_*I*_) over time (*t*) was analysed by fitting the classic one-compartment toxicokinetics model (Skip et al. 2014) to Cd concentrations in each subcellular fraction separately.

The following equations were used for the uptake phase (*t* < *t*_*c*_):

$${C_{\rm I}}\left( t \right) = {C_{I0}} \cdot {e^{ - {k_{E} \cdot t}}} + {C_{{\rm{Eu}}}}\frac{{{k_A}}}{{{k_E}}}\left( {1 - {e^{ - {k_{E} \cdot t}}}} \right),$$

and for the decontamination phase (*t* > *t*_*c*_):

$${C_I}\left( t \right) = {C_{I{t_c}}} \cdot {e^{ - {k_E} \cdot \left( {t - {t_c}} \right)}} + {C_{{\it{Ed}}}}\frac{{{k_A}}}{{{k_E}}}\left( {1 - {e^{ - {k_E}\left( {t - {t_c}} \right)}}} \right),$$

where:

$${C_{I{t_c}}} = {C_{I0}}\cdot {e^{ - {k_E} \cdot {t_c}}} + {C_{{\it{Eu}}}}\frac{{{k_{\rm A}}}}{{{k_E}}}\left( {1 - {e^{ - {k_E} \cdot {t_c}}}} \right).$$

the parameters:

– *k*_*A*_ and *k*_*E*_ indicate assimilation and elimination rate constant [day^−1^], respectively,
– *t*_*c*,_ the time of changing the food from contaminated to uncontaminated (here: 90 d of the experiment) [d],
– *C*_*0*_, initial metal concentration at *t* = 0, given in the model explicitly as the average concentration of a metal measured in ten individuals before starting the exposure (d 0) [mg kg^−1^ w.w.],
– *C*_*Eu*_ and *C*_*Ed*_, the exposure concentration in food in the uptake and decontamination phase, respectively [mg kg^−1^ d.w.].

The abovementioned model, as well as its modification, i.e. one-compartment model with three stages (Skip et al. 2014) were tested. Kinetic parameters *k*_*A*_ and *k*_*E*_ for each food type (Cd-N, Cd-A) were obtained by simultaneous fitting the equations to the data from both experimental phases using the Marquardt method. The models were fitted both to raw data and to the geometric mean concentrations calculated for each day. Geometric rather than arithmetic means were used to weaken the influence of extreme data points as we decided not to exclude possible outliers from the data-set. The *R* ^2^ for the estimated regression models were used to check how much of the total variance in data is explained (for model fitted to raw data) or how well the model depicts the general toxicokinetic pattern in time (for model fitted to geometric mean) in different fractions. All the parameters were checked for significance using 95% confidence intervals.

The effect of the food type (Cd-N, Cd-A) and the duration of exposure on Cd concentrations in each subcellular fraction were additionally tested separately for uptake and decontamination phases using a two-way ANOVA, with beetles body mass at sampling day as a covariate. The concentrations of Cd in each fraction were rank-transformed prior to ANOVA (Zar 1999). Day 0, which was common for both treatments, was excluded from the ANOVA to allow for testing interactions between the factors. After testing for both main factors and their interaction for significance (*p* ≤ 0.05), the non-significant interaction and/or non-significant covariate was removed from the model. Statistically significant differences were further analysed using Fisher’s least significant difference (LSD) test for the post hoc comparison of means. Similar analysis was done to test the effect of the food type (Cd-N, Cd-A) and the duration of exposure on Cd proportion (in percentage) in each subcellular fraction. The percentage of Cd was calculated by relating the amount of Cd retrieved from specific subcellular fraction to the total amount of the metal (i.e. the sum of Cd amounts in all the studied fractions) in the organism. Prior to ANOVA, the arc sine of the square root transformation was performed for percentages of Cd in each fraction (Zar 1999).

One-way ANOVA was used to verify the ability of the beetles to depurate down to the initial Cd concentration by comparing the Cd concentrations at days 0 and 114 for each treatment and fraction. Similarly, one-way ANOVA was used for comparison between days 0 and 90 as well as between days 90 and 114 for each treatment and fraction. One-way ANOVA with treatment as an explanatory factor was used to check for differences in Cd concentrations between treatments at the end of the uptake phase and at the end of the experiment. The contribution of Cd (in percentage) to each subcellular fraction was similarly analysed.

Although studying the effect of the food type on the beetles survival was not the aim of the study, the mortality observed during the experiment allowed us to estimate the median life time (LT_50_). LT_50_s were estimated using survival analysis with survival data censored for the beetles sampled for the kinetic studies (Kaplan and Meier 1958). The survival curves were compared between treatments with a log-rank test.

All statistical analyses were performed using the Statgraphics Centurion XVI program (StatPoint Technologies, Inc., USA).

## Results

The actual Cd concentration in artificially contaminated food (Cd-A, i.e. food made of control ground mealworms spiked with metal salt), 308 ± 12 mg kg^−1^ (mean ± standard deviation, SD), was higher than in food ‘naturally’ contaminated with Cd (Cd-N, i.e. food made of ground mealworms reared for 2 weeks on flour contaminated with 374 ± 2 mg kg^−1^ Cd), 264 ± 10 mg kg^−1^, but the difference was nonsignificant (*p* = 0.08, *N* = 3, Mann-Whitney test). In the control food the concentration was 0.17 ± 0.0015 mg Cd kg^−1^ d.w. The measured concentrations in the contaminated food were used in the toxicokinetic modelling as exposure concentrations for the uptake phase (*C*_*Eu*_), and the concentration in control food was used for the decontamination phase (*C*_*Ed*_).

The mean body mass of beetles used in the experiment was 0.0583 ± 0.00706 g with no differences between treatments (*p* = 0.3, ANOVA). The survival of the beetles did not differ between treatments (*p* = 0.3; log-rank test, survival analysis) and in all three treatments (control Cd-N and Cd-A) the LT_50_ was above 83 d (Table 1).

**Table 1:** The initial body mass [g] and the survival of the ground beetle *Pterostichus oblongopunctatus* in the control and after exposure via food made of ground mealworm (*Tenebrio molitor*) larvae either reared on Cd contaminated medium (Cd-N) or artificially spiked after grinding with CdCl_2_ solution (Cd-A); LT_50_—the median survival time (mean ± standard error), n—number of beetles in each treatment

Treatment	Body mass [g]	LT_50_ [days]	Mortality [%]
Control	0.0581 (*n* = 55)	99 ± 10.6	33
Cd-N	0.0575 (*n* = 93)	85 ± 10.4	34
Cd-A	0.0595 (*n* = 91)	83 ± 9.7	38

Individuals that had Cd concentration below the detection limit in at least one of the studied fractions (C, S1, S2 or G) were excluded from the statistical analysis. This was the case for seven beetles (all from the control treatment). We excluded the individuals rather than the particular sample (fraction) because unprecise Cd determination in one fraction would affect the calculation of the metal proportion (percentage) in each subcellular fraction for this individual.

### Metal toxicokinetics

**Fig. 1:**
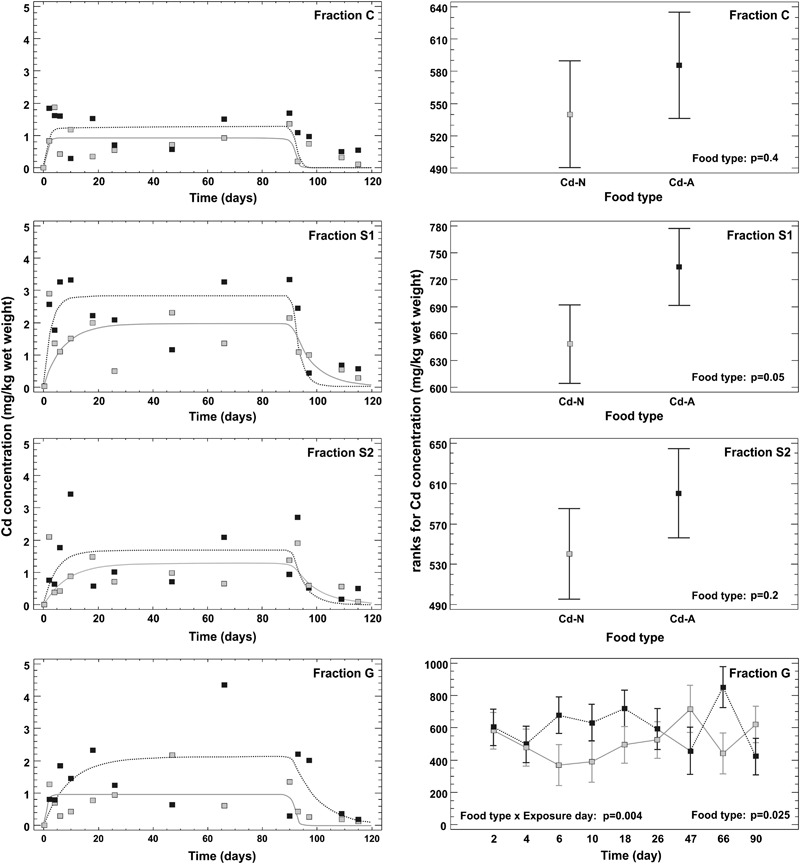
Cadmium toxicokinetic in different subcellular fractions described by the one-compartment model fitted to geometric mean concentrations (left panel) and the results of multifactor ANOVA on rank-transformed Cd concentrations showing the effect of food type on Cd concentration in subcellular fractions during the uptake phase (right panel) in the ground beetle *Pterostichus oblongopunctatus* exposed to Cd via food differing in the soluble Cd pool. The p values for the effect of food type in fractions C, S1, S2 and G as well as for the interaction between day of exposure and food type in fraction G are indicated on graphs. Cd-N—food made of ground mealworm (*Tenebrio molitor*) larvae reared on Cd contaminated medium, Cd-A—food made of ground mealworm larvae artificially spiked after grinding with CdCl_2_ solution; C—fraction combining mainly the most heavily sclerotized parts of exoskeleton (see text for more details), S1—fraction combining microsomes and lysosomes, mitochondria, metallothioneins and heat sensitive proteins, S2—fraction consisting of cell membranes, tissue fragments and intact cells, G—metal-rich granules

The initial mean internal body concentration of Cd (±standard deviation) was 0.238 ± 0.420 mg kg^−1^ wet weight (*n* = 9) with *ca*. 0.02 ± 0.033, 0.08 ± 0.211, 0.13 ± 0.391 and 0.003 ± 0.005 mg kg^−1^ in fractions C, S1, S2 and G, respectively. The one-compartment model with two phases either could not be fitted to raw data on Cd concentrations at all (this was the case for fractions C, S1 and G for Cd-N treatment) or the fit was very poor (R^2^ ranged from 0 to 9%) with nonsignificant parameters (i.e. their confidence intervals covered 0) for all fractions but two (i.e. fraction S2 for Cd-N treatment and fraction S1 for Cd-A treatment). The one-compartment model with two phases could be fitted to the geometric mean data for each fraction from both Cd treatments (Fig. 1), but the fit was poor and in most cases the estimated parameters were nonsignificant and thus could not be used for any formal comparison between fractions and/or treatments (Table 2). In fact, the model depicts the general toxicokinetic pattern satisfactorily only for Cd-A in fraction S1 with *k*_*A*_ = 0.0035 d^−1^ and *k*_*E*_ = 0.38 d^−1^ (Table 2). The three-stage model could not be fitted satisfactorily either to raw data or geometric mean data at all.

**Table 2:** Actual Cd concentrations in the food (mean ± SD) and the estimated toxicokinetic parameters (*k*_*A*_—assimilation rate constant, *k*_*E*_—elimination rate constant) with asymptotic 95% confidence intervals for the classic one-compartment model for different subcellular fractions (C, S1, S2 and G) in the ground beetle *Pterostichus oblongopunctatus* exposed via food made of ground mealworm (*Tenebrio molitor*) larvae, either reared on Cd contaminated medium (Cd-N) or artificially spiked after grinding with CdCl_2_ solution (Cd-A); *C*_*I0*_ and *C*_*114*_—internal Cd concentrations [mg kg^−1^] or percentage of Cd in each subcellular fraction (%) of beetles at the start and at the end of the experiment, respectively; C—chitin, S1—fraction combining microsomes and lysosomes, mitochondria, metallothioneins and heat sensitive proteins, S2—fraction consisting of cell membranes, tissue fragments and intact cells, G—metal-rich granules

Treatment	Cd in food	Fraction	*k*_*A*_	*k*_*E*_	*R* ^*2*^	*C*_*I0*_	*C*_90_*^a^	*C*_114_*^#a^
	[mg kg^−1^]		[day^−1^]	[day^−1^]	%	[mg kg^−1^]		
Control	0.17 ± 0.0015	C	–	–	–		0.04 ± 0.058^a^	0.02 ± 0.025^a^
		S1	–	–	–		0.56 ± 0.924^a^	0.01 ± 0.006^a^
		S2	–	–	–		0.34 ± 0.576^a^	0.002 ± 0.003^a^
		G	–	–	–		0.07 ± 0.018*^a^	0.002 ± 0.002^#a^
Cd-N	264 ± 10	C	0.004 (*NS*)	1.12 (*NS*)	26	0.02 ± 0.033	1.73 ± 1.013*^b^	0.12 ± 0.019*^#b^
		S1	0.001 (*NS*)	0.13 (*NS*)	0	0.08 ± 0.211	2.59 ± 1.737*^b^	0.27 ± 0.070*^#b^
		S2	0.0006 (*NS*)	0.12 (*NS*)	2	0.13 ± 0.391	2.13 ± 2.019*^a^	0.10 ± 0.035^#b^
		G	0.004 (*NS*)	1.1 (*NS*)	37	0.003 ± 0.005	1.83 ± 1.324*^b^	0.14 ± 0.091*^#b^
Cd-A	308 ± 12	C	0.0035 (*NS*)	0.83 (*NS*)	0		2.97 ± 3.903*^b^	0.60 ± 0.033*^c^
		S1	0.0035 (0.0002–0.007)	0.38 (0.003–0.76)	54		4.46 ± 3.314*^b^	0.57 ± 0.035*^#c^
		S2	0.001 (*NS*)	0.22 (*NS*)	20		1.20 ± 0.732*^a^	0.61 ± 0.483*^c^
		G	0.0007 (*NS*)	0.1 (*NS*)	26		0.85 ± 0.789*^b^	0.22 ± 0.156*^b^

*NS* nonsignificant (95% confidence interval for the parameter covers zero)

* Significant differences (*p* ≤ 0.05) in Cd concentration [mg kg^−1^ wet weight] between day 0 (common for all treatments) and day 90 or between day 0 and day 114 for the particular fraction

^#^ Significant differences (*p * ≤  0.05) in Cd concentration [mg kg^−1^ wet weight] between day 90 and 114

^a,^ ^b,c^ The lowercase letters mean significant differences (*p*  ≤  0.05) in Cd concentration [mg kg^−1^ wet weight] between treatments (control, Cd-N, Cd-A) for the particular fraction

### Metal concentrations in beetles

Multiple ANOVA revealed a significant effect of food type (Cd-N vs. Cd-A) on the total Cd concentration (*p* = 0.04) and concentration in fractions S1 (*p* = 0.05) and G (*p* = 0.025) in the uptake phase: in all cases higher Cd concentrations were found for Cd-A than Cd-N (Fig. 1). No effect of the day of exposure on Cd concentration was found for the studied fractions, but significant interaction between day of exposure and food type was found for the total Cd concentration (*p* = 0.027) and Cd concentration in fraction G (*p* = 0.004), indicating that the effect of food type on Cd concentration changed over time.

Multiple ANOVA for Cd concentrations in the decontamination phase revealed the significant effect of the day of exposure on total Cd concentration (*p* = 0.001) with significant body mass as a covariate (*p* = 0.016). Cadmium concentration in fraction S1 (*p* = 0.047) and S2 (*p* = 0.0004) also gradually decreased over the decontamination time, and for the latter the body mass was significant at *p* = 0.02.

Neither the total Cd concentration (*p* = 0.2) nor Cd concentration in any fraction (*p* ≥ 0.2 for all but fraction G for which *p* = 0.08) changed over the time of the experiment in the control beetles.

Total Cd concentration (*p* = 0.003) and Cd concentration in fractions C (*p* = 0.0004), S1 (*p* = 0.012), S2 (*p* = 0.04) and G (*p* = 0.0001) were higher at the end of the elimination phase (d 114) than before the exposure (d 0) for Cd-A treatment. Similar differences between d 0 and d 114 were found for fraction C (*p* = 0.009), S1 (*p* = 0.05) and G (*p* ≤ 0.0001) in Cd-N-treated beetles. The concentration of Cd in each fraction was significantly higher at d 90 than at d 0 for both Cd-A and Cd-N treatments (*p* ≤ 0.0006). Moreover, beetles offered Cd-N food had significantly lower Cd concentration at the end of the elimination phase (d 114) than at d 90 in all fractions, but similar differences between d 114 and d 90 for Cd-A treated beetles were found only for fraction S1 (*p* = 0.049). At the end of the exposure (d 90), the beetles from both Cd treatments did not differ between each other, but had significantly higher total Cd concentration (*p* = 0.001) and concentration of Cd in fractions C, S1 and G (*p* ≤ 0.04) than those from control (Table 2). The total Cd concentration (*p* ≤ 0.0001) and concentration of Cd in fractions C, S1 and S2 (*p* ≤ 0.0002) at the end of the elimination phase (d 114) differed significantly between the control, Cd-N and Cd-A treatments, with the lowest concentrations found in the control and the highest in the Cd-A treatment. The Cd concentration at the end of the elimination phase in fraction G was also significantly lower in the control than both Cd treatments (*p* = 0.0001), which did not differ from each other.

### Metal distribution among the subcellular fractions

Neither the type of food (Cd-N vs. Cd-A) nor the day of exposure affected the percentage of Cd in any fraction in the uptake phase. The contribution of Cd (in percentage) to fractions C, S1, S2 and G in the uptake phase was as follows (mean ± SD calculated for 0 < day ≤ 90): 23.4 ± 17.82, 34.7 ± 17.02, 22.2 ± 15.87 and 19.6 ± 14.36.

**Fig. 2:**
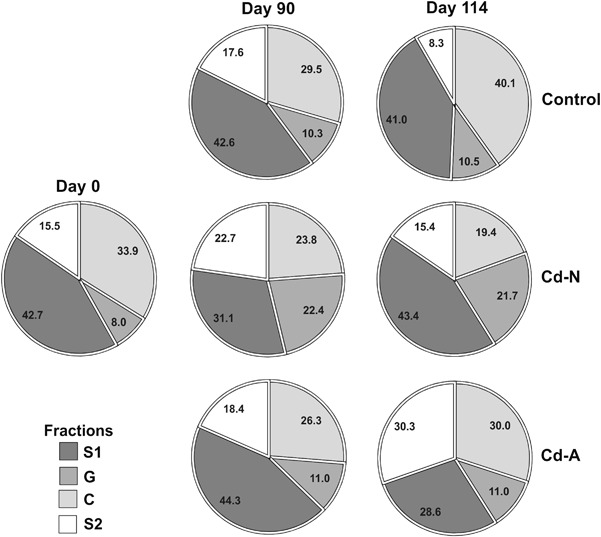
Overall patterns of Cd distribution (percentage) in the ground beetle *Pterostichus oblongopunctatus* before exposure (d 0), after 90 d of exposure to Cd via food differing in the soluble Cd pool and after 24 d of decontamination. Symbol meaning as in Fig. 1

In general, the food type did not affect the percentage of Cd in any fraction also in the decontamination phase, although a marginally significant difference (*p* = 0.06) was found for fraction S2, with a higher percentage of Cd in the Cd-N beetles. The effect of time was significant only for the percentage of Cd in fraction C (*p* = 0.0009) in this phase. Cadmium contribution to different subcellular fractions before the exposure (d 0), at the end of the uptake phase (d 90) and at the end of the experiment (d 114) is shown in Fig. 2. The percentage of Cd in any fraction did not change during the experiment in the control treatment (*p* ≥ 0.4) and did not differ between d 0 and d 114 in both the Cd-A (*p* ≥ 0.5) and Cd-N (*p* ≥ 0.1) beetles. No significant differences between d 0 and d 90 or between d 90 and d 114 were found for Cd proportion in any subcellular fraction and treatment (*p* ≥ 0.3), apart from the fraction G for which a marginally higher percentage of Cd at d 90 than at d 0 was found for Cd-N treatment (*p* = 0.06). No differences between treatments were found in the percentages of Cd in any fraction (*p* ≥ 0.3) at the end of the exposure period (d 90). Regardless of the differences between treatments found for Cd concentrations in different fractions at the end of the elimination phase (d 114), the percentages of Cd in the different fractions did not differ between treatments (*p* ≥ 0.1) with *ca*. 33 ± 22.9% of Cd found in fraction C, 39 ± 16.3% in S1, 14 ± 12.9% in S2 and 14 ±9.5% in G at the end of the experiment (means for all three treatments ± SD).

## Discussion

Cadmium occurs naturally in the environment at background concentrations in soils between 0.06 and 1.1 mg kg^−1^, but concentrations above 300 mg kg^−1^ have been found in polluted areas (Spurgeon and Hopkin 1999, Giska et al. 2014), with the highest reported Cd concentration in soil around metal smelters reaching over 1700 mg kg^−1^ (Kabata-Pendias and Mukherjee 2007). Thus, the concentrations used in our study, *ca*. 300 mg kg^−1^, can be considered realistic for some of the most polluted soils and at the same time seem high enough to study the sequestration mechanisms. Similarly to other nonessential metals, Cd has the potential for bioaccumulation in invertebrates (Peijnenburg 2002) and biomagnification along food chains (Croteau et al. 2005). Both the environmental availability of Cd and physiological constraints on uptake into an organism depend on the chemical form in which the metal is presented to the consumer. The use of the same type of food with similar metal concentration but differing in chemical partitioning of the metal is an important and valuable innovation in studies on metal toxicokinetics. In the environment, metals seldom occur as highly reactive free metal ions that have the capacity to disrupt biological systems. On the other hand, artificial food with highly bioavailable metals is usually used in the laboratory. This fact may make the extrapolation of the results from laboratory studies to the real field situation difficult, which may differ substantially in terms of metal bioavailability from the food.

### Effect of cadmium bioavailability in food on its concentrations and kinetics in different subcellular fractions

We measured Cd content in various subcellular fractions in the toxicokinetic experiment to determine to which subcellular fractions the incoming Cd is bound and at what rates the beetles sequester the metal in particular fractions. Our study revealed that the total Cd concentration and the concentration of Cd sequestered in S1 and G fractions were significantly higher in the beetles offered artificially contaminated food (Cd-A) than in those fed ‘naturally’ contaminated food (Cd-N). However, the pattern of Cd distribution over different fractions was similar in both treatments. A higher concentration of Cd in beetles from the Cd-A treatment is in accordance with our expectations, as the soluble Cd pool available to *P. oblongopunctatus* was surely higher in artificially spiked *T. molitor* larvae than in larvae reared on the Cd contaminated medium. To prepare the food ‘naturally’ contaminated with Cd, we used the larvae reared for 2 weeks on the flour contaminated with Cd at 374 mg kg^−1^. Our earlier study showed that such *T. molitor* larvae (i.e. larvae reared on flour contaminated with Cd at 380 ± 36 mg kg^−1^, so on almost identical concentration as in this study) sequestered approximately 30% of Cd in the fraction S1, which is important for transport of metals to higher trophic levels, and the contribution of Cd in the G fraction was *ca*. 30–40% (Bednarska and Świątek 2016). The higher Cd concentration in the cytosolic fraction in *P. oblongopunctatus* exposed to higher Cd availability in food is also consistent with the study on *E. fetida* that showed Cd increase in this fraction in highly contaminated soils (Conder et al. 2002). Also, the study by Calhôa et al. (2011) showed the importance of the physiological form (speciation) of the metal on its bioavailability and internal sequestration for isopods *Porcelio dilatatus*. The authors indicated that the isopods provided with a plant-based food superficially amended with ionic Cd^2+^ (Cd(NO_3_)_2_ contaminated food) assimilated *ca*. 3 times more Cd than those offered Cd biologically incorporated into the plant tissue (Cd(Cys)_2_ contaminated food). Moreover, the isopods fed with food contaminated with Cd-cysteinate had significantly more Cd distributed in the cell debris and organelles (fraction S2 in our study plus part of fraction S1) at the expense of allocation to granules (fraction G in our study) (Calhôa et al. 2011).

Because some subcellular compartments have a high affinity to metals whereas others rapidly release them, it was likely that each fraction has its own kinetic parameters (assimilation rate constant and elimination rate constant) (Li et al. 2009). In general, the beetles regulated Cd in different fractions in a similar way regardless of the food type, as no differences in kinetic parameters between fractions and/or food types were found. It has to be stressed, however, that the first-order kinetic model of treatment-specific subcellular partitioning patterns could not be satisfactorily fitted for most of the studied fractions, resulting in nonsignificant parameter values. Neither could a three-stage model (Skip et al. 2014) be tested because of the high number of parameters that need to be estimated and the high inter-individual variance of the Cd concentrations in different fractions. The high variance in Cd concentrations in the beetles throughout the experiment probably indicates the inherent variation between individuals, and was observed earlier for this species (Bednarska and Stachowicz 2013, Lagisz et al. 2005) and earthworms (Spurgeon et al. 2011). The inherent inter-individual variation in pollutant handling is probably a typical characteristic of different invertebrates under many exposure scenarios. Because of the poor fit, also the equilibrium concentrations, calculated as *C*_*Eu*_ *∙k*_*A*_/*k*_*E*_ (Skip et al. 2014), were not used to compare Cd concentrations in different fractions between treatments. Instead, we rather looked at the mean Cd concentrations for the last day of the uptake phase. Regardless of the food type, the highest Cd concentration at d 90 was found in the fraction S1, followed by the fractions S2, G and C in the Cd-N treatment, and by the fractions C, S2 and G in the Cd-A treatment.

Although the duration of exposure to a metal may affect the physiological process of metal compartmentalisation in an organism (Wallace and Lopez 1996), no clear effect of the day of exposure on Cd concentration was found for most of the studied fractions. Such results are consistent with our previous finding for *T. molitor*, the species used to prepare the food for *P. oblongopunctatus*. For *T. molitor* we found that Cd concentration in different subcellular fractions remained relatively constant during 21 d of exposure to Cd contaminated flour (Bednarska and Świątek 2016). The significant interaction between day of exposure and food type found for Cd concentration in fraction G in the present study suggests, however, that at least in this fraction the effect of food type on Cd concentration changed over time. Nevertheless, the lack of a clear trend in changes of Cd concentration in fraction G over the exposure time makes the meaning of this interaction questionable and difficult to interpret. Moreover, the small proportion of Cd found in the granules of *P. oblongopunctatus* may indicate that transferring Cd into granules is not a very effective detoxification strategy for this species. Cadmium is not known to have affinity for oxygen and therefore for pyrophosphate granules. On the other hand, as many other class B metals, Cd has a high affinity for sulphur (Hopkin 1989), so most probably sulphur-rich type B granules played a role in Cd detoxification in the beetles. It was suggested by Conder et al. (2002) that upon short-term exposure, the granules have a limited capacity for storing the incoming metal. The authors exposed the earthworm *E. fetida* to high Cd concentration (1575 mg kg^−1^) added to the artificial soil as a solution of Cd(NO_3_)_2_ for 14 d and found that Cd bound to the pellet fraction containing tissue debris and metal-rich granules (S2+G fractions in our experiment) remained stable over time (Conder et al. 2002). In our study, Cd concentration in the G fraction, although changed slightly over time in the uptake phase, remained stable after transferring the beetles to the decontamination phase. Moreover, no difference between the last day of the uptake phase (d 90) and the last day of the decontamination phase (d 114) was found for the fraction G in the Cd-A treatment which indicates on the impact of the source and form of Cd in food on the metal fate in organisms even after cessation of exposure.

### Effect of cadmium bioavailability in food on its distribution over different subcellular fractions

In both Cd treatments the S1 fraction represented the largest storage of the total accumulated Cd (31–44%) at the end of the uptake phase (d 90) followed by the fraction C (27–29%), S2 (18–23%) and G (11–22%). Such results are consistent with those for earthworms, in which Cd was mainly retrieved from the cytosolic fraction followed by debris and granules (Beaumelle et al. 2015, Li et al. 2008). In the cytosolic fraction, Cd is supposed to be mainly associated with the heat-stable protein fraction containing metallothioneins (MT) that chelate Cd (Conder et al. 2002, Vijver et al. 2004). The role of MT in the sequestration and detoxification of Cd has been well documented in terrestrial organisms (Stürzenbaum et al. 2001, Gimbert et al. 2008). The fact that Cd concentration in the cytosol was stable over time of exposure in both the Cd-A and Cd-N treatments may indicate that the internal pool of Cd bound to MT proteins was quickly saturated. Additionally, the Cd compartmentalisation in the S1 fraction, higher for the Cd-A than the Cd-N exposed beetles, may indirectly suggest the higher induction of MT in Cd-A beetles. Nevertheless, the abovementioned role of MT in sequestration of Cd in Cd-A and Cd-N beetles has to be treated with care as we did not isolate MT from S1 fraction.

The fraction C consisted of legs, head, thorax, elytra and wings of the beetles, all separated from the rest of the body before the proper compartmentalisation. This is a distinct difference between the present study and those conducted with, for example, molluscs (Wallace and Luoma 2003), earthworms (Beaumelle et al. 2015) or *T. molitor* larvae (Bednarska and Świątek 2016). Exoskeleton is often a major component of the cell debris fraction (Van Hattum et al. 1996), so to make our results comparable with those studies, the Cd amounts found in our study in fractions C and S2 should be summed up. Such a new ‘debris’ fraction has by far the highest allocation of Cd, amounting to 45–52% of all the Cd stored by the beetles. The highest allocation of Cd to cell debris (S2 fraction) followed by the S1 fraction, and finally the G fraction was found in *T. molitor* larvae exposed to Cd-contaminated flour (Bednarska and Świątek 2016). The toxicological significance of metals bound to the cell debris remains, however, undefined, as the cell debris is the most poorly defined fraction in the fractionation technique we used (Calhôa et al. 2011). It is also difficult to say if the fraction C, containing the most chitinised parts of the beetle’s body (but also the whole head and thorax deprived of only anterior gut), represents an important sink for Cd: although the beetles were washed with deionised water before being used in the subcellular fractionation, some food remnants could still be present under the elytra. Moreover, the whole head and thorax (including the tissues but without the anterior gut which was dissected from the thorax) were included in fraction C and this could affect the obtained results.

### Effect of cadmium bioavailability in food on its trophic transfer

Metal sequestration in insects is important for the transport of metals between trophic levels in food webs and thus determines the distribution of metals in ecosystems. Not all metal fractions are likely to be transferred along the food chain, and the soluble fraction of food (prey) is hypothesised to be available for assimilation by a consumer (predator), whereas metals bound to the insoluble fractions, i.e. cell walls, exoskeleton, and metal granules, are considered not to be available for predators (Wallace et al. 2003). Wallace et al. (2003) postulated that Cd associated with the organelles, heat-denatured and heat-stable proteins of the oligochaete *L. hoffmeisteri* (prey) was trophically available and assimilated at high efficiency by the grass shrimp *Palaernonetes pugio* (predator), while Cd sequestrated to metal-rich granules was much less bioavailable to the predator (Wallace et al. 2003, Wallace et al. 1998). Some authors showed, however, that also metals bound to the insoluble fraction of a prey can be mobilised via enzymes and/or digestion at acidic pH (Bragigand et al. 2004, Wallace and Luoma 2003), and the fraction of metal which is trophically available varies not only between food items and metals, but also consumers (Rainbow et al. 2011). Because gut pH may play an important role in dissolving metal-rich granules, the bioavailability of metals stored in granules by a prey is likely to be higher for a predator with a low gut pH. Probably, this was not the case in our study, as the luminal gut pH in Carabidae changes between 5.9 and 6.6 along the midgut (Terra et al. 1996), so may be too high to break down the Cd complexes or granules during the digestion of Cd-N food made of *T molitor* i.e. the food in which *ca*. 20% of accumulated Cd was stored in granules (Bednarska and Świątek 2016). The fact that the Cd stored in granules of *T. molitor* was the most probably unavailable for *P. oblongopunctatus* is also indirectly confirmed by the higher total Cd concentrations found in *P. oblongopunctatus* fed the Cd-A than Cd-N food. Also, the Cd concentration in the fractions S1 and G of *P. oblongopunctatus* in the uptake phase were higher in beetles offered artificially contaminated food (Cd-A) than in those fed with larvae reared on flour contaminated with metal (Cd-N). The latter type of food, with lower bioavailability of Cd, seems to better resemble the actual feeding habits of ground beetles. We conclude that the bioavailability of metals and, thus, their potential transfer in the trophic web vary according to the speciation of a metal in a prey, but the overall fraction of Cd transferred from different types of food seems to be similar, with at least 20% Cd taken with food being safely detoxified in granules and not (or to a small extent) available for higher trophic levels and *ca*. 35% of Cd being important for the transfer to higher trophic levels.
